# Quality of Protein Isolates and Hydrolysates from Baltic Herring (*Clupea harengus membras*) and Roach (*Rutilus rutilus*) Produced by pH-Shift Processes and Enzymatic Hydrolysis

**DOI:** 10.3390/foods11020230

**Published:** 2022-01-15

**Authors:** Tanja Kakko, Annelie Damerau, Anni Nisov, Anna Puganen, Saska Tuomasjukka, Kaisu Honkapää, Marko Tarvainen, Baoru Yang

**Affiliations:** 1 Food Chemistry and Food Development, Department of Life Technologies, University of Turku, FI-20014 Turku, Finland; tatese@utu.fi (T.K.); annelie.damerau@utu.fi (A.D.); anna.puganen@utu.fi (A.P.); saska@lapinmaria.fi (S.T.); marko.tarvainen@berner.fi (M.T.); 2 VTT Technical Research Centre of Finland Ltd., FI-02044 Espoo, Finland; anni.nisov@vtt.fi (A.N.); kaisu.honkapaa@vtt.fi (K.H.)

**Keywords:** fish valorization, isoelectric precipitation, protein quality, enzyme-assisted processing, amino acids, fatty acids, lipid classes, oxidation

## Abstract

Fractionation is a potential way to valorize under-utilized fishes, but the quality of the resulting fractions is crucial in terms of their applicability. The aim of this work was to study the quality of protein isolates and hydrolysates extracted from roach (*Rutilus rutilus*) and Baltic herring (*Clupea harengus membras*) using either pH shift or enzymatic hydrolysis. The amino acid composition of protein isolates and hydrolysates mostly complied with the nutritional requirements for adults, but protein isolates produced using pH shift showed higher essential to non-essential amino acid ratios compared with enzymatically produced hydrolysates, 0.84–0.85 vs. 0.65–0.70, respectively. Enzymatically produced protein hydrolysates had a lower total lipid content, lower proportion of phospholipids, and exhibited lower degrees of protein and lipid oxidation compared with pH-shift-produced isolates. These findings suggest enzymatic hydrolysis to be more promising from a lipid oxidation perspective while the pH-shift method ranked higher from a nutrient perspective. However, due to the different applications of protein isolates and hydrolysates produced using pH shift or enzymatic hydrolysis, respectively, the further optimization of both studied methods is recommended.

## 1. Introduction

The demand for new protein sources and the better utilization of existing resources in the food industry is increasing globally, and fish and fish products will play a crucial role in food security [1]. Fish is an excellent source of proteins and lipids, providing a balanced amino acid (AA) composition and polyunsaturated fatty acids (PUFAs), as well as minerals and vitamins. Increased demand has led to overfishing and the depletion of commonly used fish stocks, while many other fish resources are still under-utilized. For instance, in Finland, Baltic herring (*Clupea harengus membras*) (B. herring) is commercially the most significant fish, with its annual catch exceeding 100,000 tons, but most of the catch is used as feed [2]. B. herring is a small, dark-muscled fish, usually 15–20 cm in length, and has a relatively high lipid content, despite the average lipid content having decreased from 7.7% in 1988 to 4.3% in 2017 [3]. Due to the seasonal variation in fishing, fresh B. herring is not available all-year-round. Roach (*Rutilus rutilus*) is a cyprinid fish that has a low economic value and is utilized as food even to a lesser extent than B. herring. Increasing fishing of roach also has the potential to decrease eutrophication, since removing roach from the water systems directly removes nutrients, and as a protein-rich food roach contributes less to global warming compared with several commonly used protein sources, such as milk, poultry, beef, pork, eggs, and meat substitutes [4].

A potential way to increase the food use of low-value fishes is fractionation, which allows the separation, enrichment, and valorization of their proteins and lipids. Small-sized whole fish, such as a large proportion of B. herring that are too small to be filleted industrially and that currently mostly end up as feed, could also be valorized by fractionation.

Enzymatic hydrolysis has been commonly applied to recover peptides from several plant- and animal-based raw materials and by-products from meat and fish processing. Some of the resulting peptides have bioactivities, such as antioxidative and ACE I- inhibiting effects [5,6]. Several enzymes, such as pepsin, trypsin, α-chymotrypsin, pancreatin, papain, and commercial enzyme products such as Alcalase, Flavourzyme, Neutrase, and Protamex have been applied [6]. Treated fish and by-products include, for instance, salmon (*Salmo salar*) frames [7], Pacific whiting (*Merluccius productus*) [8] and its by-products [9], yellow stripe trevally (*Selaroides leptolepis*) [10], brownstripe red snapper (*Lutjanus vitta*) [11], turbot (*Scophthalmus maximus*) by-products [12], whitemouth croaker (*Micropogonias furnieri*) [13], and banded croaker (Paralonchurus brasiliensis) [13].

Alkaline or acidic extraction followed by isoelectric precipitation, also known as the pH shift, has been applied to fractionate several fishes and their by-products, such as herring (*Clupea harengus*) [14,15], bigeye snapper (*Priacanthus tayenus*) head [16], by-products of rainbow trout (*Oncorhynchus mykiss*) [17], by-products of silver carp (*Hypophthalmichthys molitrix*) [18], and by-products of salmon (*Salmo salar*), cod (*Gadus morhua*), and herring [19]. In the process, proteins are first solubilized using an acid or base to increase their positive or negative net charge, inducing protein–protein electrostatic repulsion, and increasing protein–water interactions. After solubilization, pH is adjusted to the isoelectric point of the protein, where proteins have a net charge close to zero, causing precipitation. The pH shift process has the advantage that the proteins are not significantly cleaved or do not have to be subjected to a heat treatment and hence retain certain functional properties, such as gelation [15].

The quality of the resulting protein isolate or hydrolysate determines its applicability. The composition of amino acids, as well as the digestibility and bioavailability of the protein, is essential in terms of nutritional value [20]. Controlling lipid oxidation is a major challenge in the case of both pH-shift processing [21] and enzymatic hydrolysis [22]. The polyunsaturated lipids in fish are highly susceptible to oxidation, especially due to the presence of pro-oxidants, mincing and high temperatures or extreme pH conditions used in the extraction. Lipid oxidation further promotes the oxidation of proteins and reduces sensory quality and nutritional value [23,24]. The potential application is limited for a protein isolate or hydrolysate with strong fishy odor and flavor, mostly derived from lipid oxidation [19,25,26]. Not only lipid content, but also composition may affect the development of lipid oxidation, since the susceptibility of lipids to oxidation is dependent on the degree of unsaturation [23]. Phospholipids (PLs) are considered to be more susceptible than triacylglycerols (TAGs) due to their high degree of unsaturation as well as their presence in cell membranes [27]. Previous studies on enzymatic hydrolysis or the pH-shift processing of fish have reported varying lipid contents and levels of lipid and protein oxidation in the resulting protein isolates [18,21,22,28]. Despite relatively high lipid contents reported [9,14,17,18], there is little information on the composition of lipids retained in the protein isolates from previous studies.

The aim of this work was to study the potential of enzymatic hydrolysis and pH-shift processing in the valorization of proteins/peptides from small-sized, whole B. herring and roach. Therefore, the study focused on the quality of proteins/peptides and remaining lipids in isolated proteins and peptides produced by two different pH-shift methods (alkaline vs. acidic) and enzymatic hydrolysis using three different commercial endoproteases from B. herring and roach. The amino acid composition of produced isolates and hydrolysates was analyzed to estimate their nutritional value. The composition and quality of the lipids remaining in the isolates and hydrolysates was studied by analyzing fatty acid and lipid class composition. In addition, protein carbonyls, peroxide values and lipid-oxidation-derived volatile compounds were analyzed as markers of oxidation.

## 2. Materials and Methods

### 2.1. Materials

#### 2.1.1. Raw Materials and Processing

Freshly caught B. herring (caught in September from the Archipelago Sea) with no preprocessing and descaled roach (caught in June from the Bothnian Sea) were bought from AK Foods Arvo Kokkonen Oy (Finland). The B. herring used as raw material were of smaller size (<17 cm) than the portion of the catch directed for food use. Roach were on average 20 cm long. Both fish batches were ground as whole within 24 h after catching and stored frozen at −22 °C until being processed (3 and 1 months, in the case of roach and B. herring, respectively). Protein isolates and hydrolysates were produced at the VTT Technical Research Centre of Finland (Espoo, Finland), and the processes have previously been described in detail by Nisov et al. [29]. Briefly, pH-shift fractionation was performed using both acidic (A, pH 2.5) and alkaline (B, pH 11.5) solubilization, followed by precipitation at pH 5.2. Homogenized fish was mixed with tap water in a 1:4 ratio (1 part fish + 3 parts water), and 6 M or 1 M (for fine adjustment) hydrochloric acid or sodium hydroxide was added under continuous mixing (75 rpm) until target pH was reached. After reaching the solubilization pH, mixing was continued for 15 min to allow enough time for solubilization. Phase separation after solubilization and precipitation was achieved by centrifugation at 4000× *g* for 15 min. In the case of B. herring, which contained more lipids, the centrifugation of the supernatant after protein solubilization was repeated to enhance lipid removal. The mixture was kept at 4 °C during solubilization and precipitation, but as a precaution to prevent microbial spoilage, after pH adjustment to 5.2 and prior to separation of precipitated proteins, the suspension was heated until 75 °C was reached and was held for 1 min, after which the suspension was again cooled down to 4 °C (Supplementary Figure S1). The pH shift itself is a mild non-thermal “pasteurization” due to a high or low pH used [30,31], and hence pasteurization by heat is often not necessary. In the present study, the heating step was, however, included, since whole ungutted fish were used, and the presence of intestinal microbes and enzymes of the fish could have been detrimental to the stability of the protein isolates.

Enzymatic hydrolysis was conducted using three different endoproteases, Protamex (Novozymes, Bagsværd, Denmark), Neutrase (Novozymes), and Corolase 7089 (AB Enzymes, Darmstadt, Germany). Homogenized fish was diluted in tap water in 1:2 ratio (1 part fish + 1 part water). Enzymes were added at a dose of 11.4 nkat/g. Hydrolysis was conducted at 50 °C for 30 min under continuous mixing, after which enzymes were inactivated at 75 °C for 15 min. After cooling the mixture to 4 °C, centrifugation at 4000× *g* for 15 min was applied to separate insoluble matter and lipids, and this step was repeated for the supernatant in the case of B. herring. All protein fractions (from pH shift and enzymatic fractionation) were freeze-dried. All treatments were performed in triplicate. A detailed graphical description of both processes can be found in Supplementary Figure S1. The protein isolates and hydrolysates and raw materials analyzed in this study have been previously characterized for their protein and lipid content [29] and the results are cited in Table 1. Freeze-dried isolates and hydrolysates were stored at −80 °C until analysis.

#### 2.1.2. Reagents and Standards

L-amino acid standards, norvaline, sarcosine, sodium dihydrogen phosphate (NaH_2_PO_4_), hydrochloric acid, sodium hydroxide, guanidine hydrochloride, 2,4-dinitrophenylhydrazine, iron (II) chloride, iron (III) chloride, ammonium thiocyanate, *ortho*-phthalaldehyde, 9-fluorenylmethoxycarbonyl chloride, iodoacetic acid, 3-mercaptopropionic acid, boric acid (H_3_BO_3_), trichloroacetic acid, and MS-grade ammonium acetate were from Sigma-Aldrich (Darmstadt, Germany). Standards for fatty acid analysis included Supelco 37 Component FAME mix (Supelco, St. Louis, MO, USA), 68D (Nu-Check-Prep, Elysian, MN, USA), and GLC-490 (Nu-Check-Prep, Elysian, MN, USA). Standards for the lipid class analysis were oleic acid, oleoyl monoacylglycerol, dioleoyl diacylglycerol, triolein, dioleoyl phosphatidylcholine, cholesteryl oleate and ethyl docosahexaenoic acid (Larodan, Solna, Sweden). All solvents used were at least HPLC grade. Water used was ultra-pure water (ELGA LabWater, High Wycombe, UK).

### 2.2. Amino Acid Analysis

Amino acids were analyzed using reverse-phase high-performance liquid chromatography with fluorescence detection (RP-HPLC-FLD). Prior to analysis, the samples were hydrolyzed to amino acids using acid and alkaline hydrolysis [32]. Tryptophan was quantified after alkaline hydrolysis; other amino acids after acidic hydrolysis. For acid hydrolysis, 5 mL of 6 M hydrochloric acid was added to 90 mg of protein sample weighed in a glass tube. The tubes were flushed with nitrogen to exclude oxygen and placed in a 110 °C oven for 20 h. Cysteine was only partially recovered with this hydrolysis method. Alkaline hydrolysis was conducted similarly, except for the addition of 4.2 M sodium hydroxide instead of hydrochloric acid. All hydrolysates were diluted 1:10 using ultrapure water (1 part hydrolysate + 9 parts water), filtered using 0.2 µm regenerated cellulose syringe filters (Lab Logistics Group GmbH, Meckenheim, Germany), and stored frozen at −80 °C until analysis. Acid hydrolyses were performed in duplicate for each of the three process replicates, yielding six replicates in total. Alkaline hydrolyses were performed once for each process replicate. Before derivatization, hydrolyzed samples were further diluted 1:10 in ultrapure water and norvaline and sarcosine were added as internal standards. Amino acids were derivatized according to Henderson et al. [33], with the exception of the addition of iodoacetic acid (5 mg/mL in borate buffer) to derivatize cysteine residues [34].

A Shimadzu (Shimadzu Corporation, Kyoto, Japan) LC-20AD system coupled to a SIL-20AC autosampler, column oven CTO-20AC, communication module CBM-20A, and RF-20Axs fluorescence detector was used for amino acid analysis, along with a Zorbax Eclipse AAA column (150 × 4.6 mm, 3.5 μm; Agilent Technologies, Santa Clara, CA, USA) attached to a precolumn. Chromatographic separation and the detection of amino acids were conducted according to the protocol provided by Henderson et al. [33]. Briefly, chromatographic separation was achieved using a gradient of mobile phase A (40 mM NaH_2_PO_4_, pH 7.80 ± 0.01) and B (45/45/10, *v/v/v*, acetonitrile/methanol/water). Mixtures of commercial amino acid standards were used to prepare an 8-point calibration curve, and amino acids in samples were quantified using calibration curves of external standards and corrected by internal standards.

### 2.3. Extraction and Analysis of Lipids

Lipids were extracted for analyzing their quantity and composition using the method by Folch et al. [35] with a slight modification; a more concentrated (8.8%) solution of potassium chloride solution was utilized for phase separation. Extraction was performed once for each process replicate.

Fatty acids were analyzed using gas chromatography (GC) with a flame ionization detector (FID) as methyl esters (FAMEs) prepared with an acid-catalyzed method [36]. Briefly, lipid samples and internal standard (PC19:0; 1,2-dinonadecanoyl-*sn*-glycero-3-phosphatidylcholine, Larodan, Solna, Sweden) were methylated overnight at 50 °C using acetyl chloride/methanol (1/1, *v/v*). After the addition of potassium carbonate and hexane, tubes were vortexed and centrifuged at 1000 rcf for 3 min, and hexane phase containing FAMEs was collected. Each lipid extract was methylated twice, yielding 6 replicates for each sample type. GC-FID analysis was conducted as previously reported by our group [37]. The peaks were identified using external standards and quantified using internal standard and correction factors determined with standard mixtures.

Lipid classes were determined using ultra-high-performance liquid chromatography with electrospray ionization and mass spectrometer (UHPLC–ESI–MS) as previously described by our group [38]. Briefly, chromatographic separation was achieved using a gradient of 10 mM ammonium acetate in MeOH:water (1/1, *v/v*) (mobile phase A) and 10 mM ammonium acetate in 2-propanol:water (1000/1, *v/v*) (mobile phase B) (0–33 min A:99%, 33–36 min A:1%, and 36–38 min A:99%) at a flow rate of 0.250 mL/min on a Waters Cortecs UPLC C18 1.6 μm, 2.1 × 100 mm column (Waters co., Milford, MA, USA). Mass scans between 100 and 1500 *m*/*z* were performed in both positive and negative ionization modes. Prior to the analysis, the concentration of lipids in all extracts was adjusted to 0.5 mg/mL in chloroform:methanol (2:1, *v/v*). Lipid classes in the raw materials were semi-quantified based on peak area and standards with predetermined response factors [38]. 

### 2.4. Determination of Peroxide Values, Protein Carbonyls and Volatiles

Peroxide values (PVs) were determined according to a ferric thiocyanate method as described by [39]. PV measurement was performed in duplicate from each lipid extract (process replicate). Protein carbonyls were quantified as an indicator of protein oxidation. The widely used protein carbonyl method by Levine et al. [40] was used to quantify protein carbonyls in the pH-shift-processed isolates. Briefly, protein isolates were dissolved in 6 M guanidine hydrochloride, incubated in the dark (RT) for 60 min with 2,4-diphenylhydrazine (DNPH), after which proteins were precipitated using ice-cold trichloroacetic acid. Protein pellet was washed thrice with ethanol:ethyl acetate (1:1) to remove excess DNPH, and after re-solubilization of protein pellet in guanidine-HCl, the absorbance was measured at 374 nm against a blank (protein solution without added DNPH). Protein contents in samples (after precipitation and resolubilization) were quantified by measurement at 283 nm and comparison with a standard curve established by fish protein with known protein concentration.

Enzymatically produced hydrolysates could not be quantified using the before mentioned protocol, since it involves the precipitation of proteins using trichloroacetic acid, which was impossible for the enzymatically processed samples due to their high degree of hydrolysis and solubility [29]. Protein carbonyls in enzymatically produced hydrolysates were therefore quantified using a simplified method by Mesquita et al. [41], which does not require protein precipitation and the removal of unbound DNPH, because the absorbance of labeled protein carbonyls is shifted to 450 nm due to the alkaline conditions used in the assay. Mesquita et al. [41] reported that the simplified method showed high correlation with the original method by Levine et al. [40], but due to the use of different methods the carbonyl results of isolates and hydrolysates were not compared with each other.

Volatile compounds in protein isolates and hydrolysates were extracted using headspace solid-phase microextraction (HS-SPME) and analyzed using GC–MS. For the analysis, 0.5 ± 0.01 g of sample was weighed in 20 mL SPME vials, and 3 mL of water was added. In the case of the raw materials, B. herring and roach, 3.0 ± 0.025 g of ground fish was weighed, and no water was added. All vials were flushed with nitrogen to limit oxidation during the analysis. HS-SPME–GC–MS analysis was conducted as described previously by our group [37]. In brief, the extraction of volatiles was conducted at 40 °C for 30 min after incubation at 40 °C for 20 min, using a DVB/CAR/PDMS-fiber (50/30 μm film thickness; Supelco, St. Louis, MO, USA). GC–MS analysis consisted of the desorption of volatiles for 6 min at 240 °C, and their separation using Supelco SPB-624 column (60 m × 0.25 mm i.d., 1.4 μm film thickness; Supelco, St. Louis, MO, USA). MS was operated in electron ionization mode and ions were scanned in the range 50–300 amu.

### 2.5. Statistical Analysis

Statistical comparisons were performed using one-way ANOVA and Tukey’s HSD test, or the independent-samples T test with SPSS (IBM SPSS Statistics, version 25.0.0.1, IBM, New York, NY, USA). ANOVA was used when comparing multiple samples to each other; T test was used in the case of lipid class data, when the protein isolates or hydrolysates were compared with the raw material. The difference between samples was considered statistically significant if *p*-value was below 0.05.

## 3. Results

### 3.1. Amino Acid Composition

The amino acid contents in protein isolates and hydrolysates, as well as raw materials, are presented in Table 2. There was little variation in the amino acid contents within isolates/hydrolysates produced using the same type of fractionation method (pH shift or enzymatic hydrolysis) with few statistically significant differences. In the case of roach isolates produced using acidic and alkaline pH shift, the alkali-processed isolate had a statistically significantly higher (*p* < 0.05) content of tryptophan (13.5 mg/g protein) and lower content of arginine (55.0 mg/g protein) compared with acidic isolate (11.7 and 57.7 mg/g protein, respectively). However, in the case of B. herring, the alkaline isolate had a statistically significantly higher content of arginine (63.8 mg/g protein) and lower content of glutamine + glutamic acid (125.5 mg/g protein) than the acid-extracted isolate (59.7 and 131.5 mg/g protein, respectively). In the case of enzymatically produced hydrolysates there were only minor differences in the amino acid composition depending on the enzyme used.

However, the use of different types of extraction methods (hydrolysis vs. pH shift) was reflected in the amino acid composition. Most notable differences were observed in the contents of asparagine + aspartic acid, glycine, hydroxyproline, tyrosine, isoleucine, phenylalanine, tryptophan, and valine. Protein isolates produced using pH shift had a significantly lower content of glycine (28.5–31.0 mg/g protein) compared with that of the fish raw material (38.0–42.4 mg/g protein), or enzymatically produced hydrolysates (44.8–51.9 mg/g protein). This finding is in line with studies by Abdollahi and Undeland [19] and Marmon and Undeland [14], where protein isolates produced by pH-shift processes from herring had lower glycine contents compared with the level in herring itself. The authors of the latter study hypothesized that this could be either due to loss of protein(s) rich in glycine or the fact that glycine is one of the most abundant free amino acids in herring, and is likely discarded with the water phase during the second separation of the process. In the present study, enzymatically produced hydrolysates showed even higher contents of glycine compared with the levels in raw fish, and since these hydrolysates are collected with the water phase, it can be expected that the loss of free amino acids occurs to a lesser extent during the enzymatic fractionation.

The content of hydroxyproline was expectedly lower in pH-shift-processed isolates (1.3 mg/g protein in both acid and alkali-extracted isolates of roach, and 2.0 and 2.1 mg/g protein in acid and alkali-extracted isolates of B. herring, respectively) compared with contents in the raw materials (5.3 and 6.7 mg/g protein in roach and B. herring, respectively) and in the enzymatically produced hydrolysates (10.6–11.9 and 8.7–9.1 mg/g protein, in the case of roach and B. herring hydrolysates, respectively). Hydroxyproline is almost exclusively found in collagen, which is efficiently removed in the precipitate of the first centrifugation of the pH shift [42]. On the other hand, higher hydroxyproline levels in the hydrolysates indicate that the use of proteases, or heating applied during hydrolysis, resulted in the extraction of collagen. Collagen is also rich in glycine and proline [42] and hence the removal of collagen may also have contributed to lower glycine levels in protein isolates produced by pH shift. The content of proline in pH-shift-processed isolates was lower than that in enzymatically produced hydrolysates in the case of roach (31.8 and 32.2 in isolates produced using acidic or alkaline pH shift vs. 38.4–40.2 mg/g protein in hydrolysates produced using Protamex, Neutrase, and Corolase, respectively), but not in the case of B. herring.

The acidic and alkaline pH-shift-processed isolates from both fishes were more abundant in certain essential amino acids (EAA), such as isoleucine and phenylalanine. The essential to non-essential amino acid (NEAA) ratios were statistically significantly (*p* < 0.05) higher in the pH-shift isolates (0.84, and 0.85, in acid- and alkali-extracted isolates of roach, and 0.85 in both acid- and alkali-extracted isolates of B. herring, respectively) compared with the raw material (0.79 in the case of roach and 0.77 in the case of B. herring).

Studies by Marmon and Undeland [14], Zhong et al. [18], and Abdollahi and Undeland [19] also reported high EAA contents for protein isolated using pH shift from herring and by-products of rainbow trout, silver carp, salmon, cod, and herring. The statistically significantly higher (*p* < 0.05) EAA to NEAA ratio of proteins extracted using pH shift (0.84–0.85) compared with the ratio in the fish raw material (0.79 and 0.77 in roach and B. herring, respectively) in the present study can likely be attributed to the removal of collagenous material and therefore the higher loss of the non-essential amino acids glycine, proline, and hydroxyproline than essential amino acids. The EAA to NEAA ratios in enzymatically produced hydrolysates (0.69, 0.70, 0.65 in Protamex, Neutrase, and Corolase hydrolysates of roach, and 0.67 in the case of all B. herring hydrolysates), on the other hand, were statistically significantly lower compared with the respective raw materials. Studies on enzymatically produced fish protein hydrolysates have generally reported amino acid contents meeting nutritional requirements for adults (FAO/WHO/UNU, 2007). For instance, Klompong et al. [10] reported increased EAA to NEAA ratios after the hydrolysis of yellow stripe trevally (*Selaroides leptolepis*) using Alcalase and Flavourzyme. Sartesh et al. [43] reported an increase in EAAs from 48% in the muscle to 58% of total amino acids in the hydrolysate of Sind sardine muscle, produced with pepsin. Some studies have, however, reported losses in tryptophan and other EAAs [9,44], as was the case in the present study. The observed decrease in EAA to NEAA ratio in hydrolysates compared with raw materials in the present study may be explained by loss of hydrophobic EAAs, valine, leucine, isoleucine, phenylalanine, methionine, and tryptophan to the sediment during centrifugation.

All protein isolates and hydrolysates generally complied well with the FAO/WHO/UNU [20] recommendations for adults. In enzymatically produced hydrolysates of roach and B. herring, valine content was 31.8–32.7 and 30.1–30.7 mg/g protein, respectively, which was slightly below the recommended level of 39 mg/g protein, making valine the limiting amino acid. 

### 3.2. Fatty Acid Composition

The fatty acid compositions of the lipids retained in protein isolates and hydrolysates from roach and B. herring are presented in Table 3. In the case of roach samples, all isolates and hydrolysates contained statistically significantly (*p* < 0.05) more saturated fatty acids (SFAs)/total fatty acids (25.1–27.6 mg/100 mg total fatty acids) compared with the raw material (23.1 mg/100 mg total fatty acids). With the exception of the acidic isolate, all other isolates/hydrolysates contained statistically significantly less polyunsaturated fatty acids (PUFAs) (25.4–29.1 mg/100 mg total fatty acids) and more monounsaturated fatty acids (MUFAs) (45.8–46.9 mg/100 mg total fatty acids) compared with the raw material (34.0 mg PUFAs and 42.8 mg MUFAs of 100 mg total fatty acids).

The fatty acid composition in protein isolates and hydrolysates of B. herring showed a similar trend in terms of SFAs, with all isolates and hydrolysates containing a statistically significantly higher relative content (26.6–35.3 mg/100 mg total fatty acids) compared with the raw material (25.5 mg/100 mg total fatty acids). The acidic and alkaline pH-shift isolate in particular contained a considerably higher proportion of SFAs (34.2 and 35.3 mg/100 mg total fatty acids), but also a lower proportion of MUFAs (32.1 and 37.5 mg/100 mg total fatty acids) compared with the raw material (25.5 mg SFAs and 41.4 mg MUFAs/100 mg total fatty acids).

There were also significant differences in the composition of fatty acids within the same processing type (pH shift or enzymatic hydrolysis). For instance, in the case of roach protein hydrolysates, the one produced using Protamex had a statistically significantly lower relative content of SFAs and higher content of PUFAs than those produced using Neutrase and Corolase (24.9 vs. 27.0 and 27.6 mg/100 mg total fatty acids in the case of SFAs and 28.7 vs. 26.7 and 25.4 mg/100 mg total fatty acids in the case of PUFAs, respectively). On the other hand, acid- and alkali-treated protein isolates from both fishes had similar levels of SFAs, but alkali-solubilized protein isolates of both roach and B. herring had significantly more MUFAs (45.8 vs. 41.2 and 37.5 vs. 32.1 mg/100 mg total fatty acids) and less PUFAs (29.1 vs. 33.5 and 27.2 vs. 33.8 mg/100 mg total fatty acids) compared with acid-treated isolates of the same raw material. The varying amount of PUFAs could be related to different levels of loss through oxidation, and the possible differential distribution of lipid classes during fractionation. Interestingly, pH-shift isolates, especially acidic isolate from B. herring, had a significantly higher content of docosahexaenoic acid (22:6 n-3, DHA) (14.4 mg/100 mg total fatty acids) compared with raw materials (8.6–10.5 mg/100 mg total fatty acids) and enzymatically produced hydrolysates (6.4–9.2 mg/100 mg total fatty acids). Acidic isolates of both B. herring and roach also had a slightly higher relative content of eicosapentaenoic acid (20:5 n-3, EPA) compared with other protein isolates and hydrolysates (7.4 vs. 6.4–7.1 in the case of roach and 7.0 vs. 5.5–6.6 mg/100 mg total fatty acids in the case of B. herring), although the difference was not statistically different in the case of roach acidic isolate (7.4 mg) vs. Protamex hydrolysate (7.1 mg). The observed differences in fatty acid composition could be related to the differences in lipid classes remaining in either protein isolates or hydrolysates, e.g., PLs have been shown to display a higher level of unsaturation compared with TAGs.

In general, the composition of lipids remaining in the protein/peptide fraction, extracted using pH shift or enzymatic hydrolysis, has not been studied, despite that in some occasions relatively high lipid contents have been reported [9,14,17,18]. Wu et al. [45] reported the total contents of PUFAs in protein isolates produced by alkaline pH shift, as well as in the raw materials, backbones of herring, cod and salmon. The amount of PUFAs/kg raw material or protein was expectedly lower in the protein than in the raw material in the case of herring and salmon backbones, as the total content of lipids was also lower. However, the proportion of PUFAs of total lipids increased after the extraction from herring, but decreased after the extraction of salmon backbones. In a study by Abdollahi et al. [46], protein isolate produced from herring backbones by alkaline pH-shift contained a considerably higher percentage of PUFAs compared with the raw material (43% vs. 24% of total fatty acids). In the present study, the proportion of PUFAs was statistically significantly (*p* < 0.05) lower in the alkali-extracted isolates of both roach and B. herring, when compared with the raw material, whereas no statistically significant differences were observed between acid-extracted isolates and raw materials.

The present study suggests that not only the lipid content but the fatty acid composition are considerably influenced by the processing conditions. The fatty acid composition of the lipids retained in protein isolates or hydrolysates will affect their susceptibility to oxidation, as PUFAs are more prone to oxidation than SFAs. However, in terms of nutritional value, a high content of long-chain PUFAs, such as EPA and DHA, may be desirable.

### 3.3. Lipid Classes

**Figure 1:**
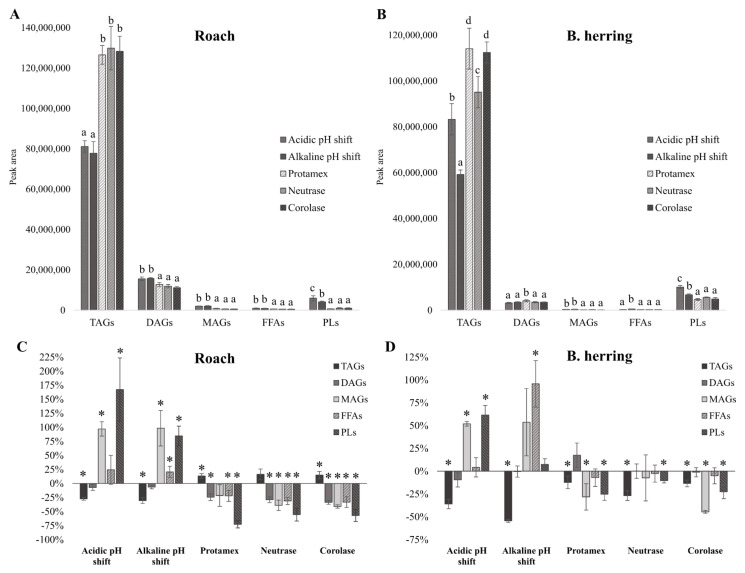
Sum peak areas of triacylglycerols (TAGs), diacylglycerols (DAGs), monoacylglycerols (MAGs), free fatty acids (FFAs), and phospholipids (PLs) in protein isolates and hydrolysates of roach (**A**) and B. herring (**B**), and relative changes in lipid class peak areas in lipids of protein isolates and hydrolysates compared with the fish raw material (**C**,**D**). Different letters or an asterisk (*) within each lipid class indicate a statistically significant difference (*p* < 0.05). Peak areas in an equal content of lipids were determined using liquid chromatography mass spectrometry.

The composition of the lipid classes retained in the protein isolates and hydrolysates, and the lipid classes in the fish raw materials were assessed semi-quantitatively by analyzing the lipid classes using LC–MS. Figure 1 presents the sum peak areas of the detected lipid classes of lipids extracted from the protein isolates and hydrolysates, as well as relative changes in lipid composition compared with the lipid composition of the raw material. All peak areas are presented in an equal amount of lipids.

Linko et al. [47] reported B. herring lipids to comprise 53.5–91.2% TAGs, 7.6–41.3% PLs, 0–2.2% diacylglycerols (DAGs), 0.6–1.9% cholesterol, and 0.1–1.2% free fatty acids (FFAs), the proportions varying depending on the season. The highest relative content of TAGs was observed in fish caught in the late autumn when the PL content was at its lowest, whereas the lowest relative TAG content was observed in the fish caught in the summer and the PL content was at its highest. Based on semi-quantitative data, the lipids of B. herring used as raw material contained approximately 78.3 ± 0.8% TAGs, 18.8 ± 0.7% PLs, 2.1 ± 0.2% DAGs, 0.2 ± 0.0% monoacylglycerols (MAGs), and 0.6 ± 0.1% FFAs. The B. herring was caught in September and hence the results are in line with the study of Linko et al. (1985) [47]. The lipids in roach raw material, on the other hand, contained 77.9 ± 1.0 TAGs, 7.7 ± 0.5 PLs, 11.7 ± 0.5 DAGs, 0.7 ± 0.1 MAGs, and 2.0 ± 0.1 FFAs, having less PLs and more DAGs, FFAs, and MAGs of total lipids, when compared with B. herring. Higher relative contents of DAGs, MAGs, and FFAs can be partly explained by the difference between the two species but possibly also by the hydrolysis of TAGs or PLs during the frozen storage of minced roach (3 months at −22 °C) prior to processing. Nisov et al. [29] reported findings that strongly indicated high endogenous protease activity in the roach raw material, as opposed to B. herring. It is possible that endogenous lipase activity may also be higher in roach compared with B. herring, and thus explain the relatively high proportions of DAGs, MAGs, and FFAs observed in roach raw material.

Classes of lipids in fish protein isolates and hydrolysates have seldom been reported, apart from some studies that reported the PL content [15,24]. According to the results of the present study, there were significant differences in the composition of lipids retained in the protein isolates or hydrolysates obtained with different processes (Figure 1). In the case of roach isolates/hydrolysates, the lipids in alkaline and acidic pH-shift-processed isolates contained statistically significantly (*p* < 0.05) more PLs/total lipids compared with enzymatically produced hydrolysates. Acid- and alkali-extracted roach isolates also exhibited 167% and 85%, respectively, higher peak areas of PLs compared with the lipids in the roach raw material. Similarly, pH-shift-processed protein isolates from B. herring, contained a statistically significantly higher proportion of PLs compared with enzymatically produced hydrolysates of the same raw material (Figure 1B). However, when compared with the B. herring raw material, a statistically significantly higher peak area of PLs was only seen in the case of the acidic protein isolate (Figure 1D). The observed increase in DHA (22:6 n-3) in the acidic protein isolate of B. herring may have been due to the increased PL content, as membrane PLs are rich in PUFAs [23]. Membrane PLs are suggested to be the main substrates of muscle tissue lipid oxidation [15] and therefore their removal during protein extraction is often desired. The accumulation of PLs in the pH-shift processed isolates may have contributed to the higher degree of oxidation, as further discussed in Section 3.4. Membrane lipids, consisting mostly of PLs [23], are suggested to be at least partly removed during the first centrifugation step of the pH-shift process, especially when high-speed centrifugation is used. In a study by Undeland et al. [15], a high-speed centrifugation step (18,000× *g*, 20 min) was seen to reduce the total lipid and PL contents in acidified herring (after solubilization of proteins in acidic pH shift) by 70% and 45%, respectively, by comparing the content of lipids/g of protein. Interestingly, in the same study it was seen that while a longer holding time (65–125 min) between acidification and the first centrifugation step drastically increased overall lipid removal, a holding time of 125 min, compared with 5 min, decreased PL removal from 30% to 0%. It should be noted, however, that when 45% of PLs were removed, the reduction in total lipids was 70%, meaning that after the process the protein isolate contained a higher proportion of PLs of total lipids than the fish raw material. In the present study, the total lipid content of alkaline protein isolate from roach was even higher on a dry weight basis compared with the raw material (19.4 vs. 17.0%), and since the peak area of PLs in lipids of the alkaline isolate was 85% higher than in the roach raw material, the total content of PLs (on d.w. basis) was also higher. In this study, after reaching the target pH values in acid and alkaline solubilization, mixing of the homogenate was continued for a further 15 min to allow more proteins to solubilize. The 15 min delay before centrifugation and centrifugation at low speed (4000× *g*, 15 min) could have contributed to the accumulation of PLs in the pH-shifted protein isolates from roach.

On the other hand, the enzymatic fractionation of roach and B. herring significantly reduced the proportion of PLs of total lipids (Figure 1), despite the same centrifugation force and time applied as in the case of the pH-shift process. The reduction was especially prominent in the case of roach; lipids in enzymatically produced hydrolysates contained 56–73% less PLs compared with the lipids in the raw material. The hydrolysates produced using Protamex, Neutrase, and Corolase from roach also contained less DAGs, MAGs, and FFAs of total lipids compared with the raw material, whereas the amount of TAGs was higher compared with the raw material. B. herring hydrolysates, compared with roach hydrolysates, showed smaller differences to the B. herring raw material in terms of lipid composition. On the other hand, there were, interestingly, some differences between B. herring hydrolysates produced with different enzymes. Lipids in hydrolysate produced using Neutrase contained statistically significantly less TAGs compared with hydrolysates produced by Protamex and Corolase.

The pH-shift-processed isolates from both roach and B. herring contained significantly less TAGs of total lipids compared with the hydrolysates. The lower content of TAGs may be related, in addition to the difference in extracted lipids, to hydrolysis by lipases or changes due to oxidation, and was also accompanied by a higher proportion of FFAs in the pH-shift protein isolates. The alkali-extracted protein isolate of B. herring in particular also contained significantly more FFAs and was found to have the highest PV and peak areas of volatile lipid oxidation indicators (Section 3.4) compared with acid-extracted protein isolate and hydrolysates of B. herring. Extremely alkaline conditions, in addition to lipases, induce the hydrolysis of TAGs [48]. Hydrolysis and oxidation are connected. Hydrolyzed lipids may be more susceptible to oxidation, whereas oxidized lipids may be more susceptible to hydrolysis by lipases [27]. Yarnpakdee et al. [24] analyzed the content of PLs and FFAs in muscle of Nile tilapia (*Oreochromis niloticus*) prior to hydrolysis with Alcalase. During an 18-day storage period of the muscle on ice, they observed a decrease in PLs and increase in FFAs, which coincided with an increase in PV and thiobarbituric acid reactive substances (TBARS). On the other hand, it was reported by two separate studies that the addition of free fatty acids in a washed turkey model, and the hydrolysis of phospholipids using phospholipase A2 in washed cod muscle inhibited hemoglobin-mediated lipid oxidation [49,50]. The oxidation of lipids may also lead to their polymerization, but possible polymerized lipids were not detected with the LC–MS method used in this present study.

### 3.4. Peroxide Values, Protein Carbonyls and Oxidation-Related Volatile Compounds

**Figure 2:**
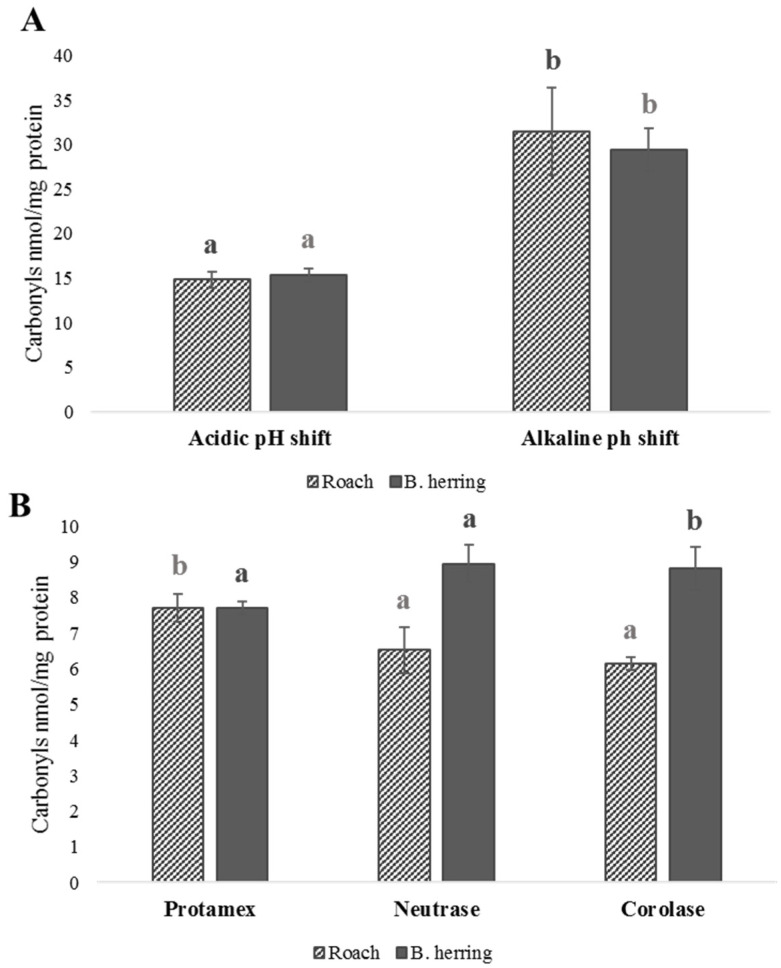
Protein carbonyls (nmol/mg protein) in protein isolates produced using pH shift (**A**), quantified using the original carbonyl method [40], and in enzymatically produced hydrolysates (**B**), analyzed using a modified method by Mesquita et al. [41]. Different letters within the same raw material indicate a statistically significant difference (*p* < 0.05).

**Figure 3:**
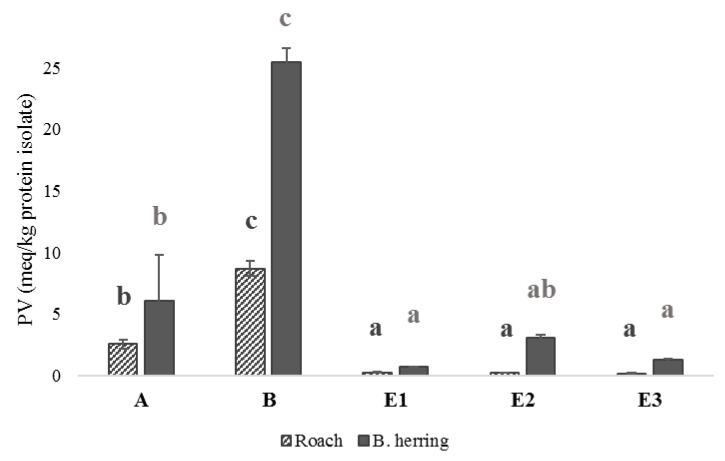
Peroxide values (meq/kg protein isolate) in protein isolates produced using acidic or alkaline pH shift and hydrolysates produced using Protamex, Neutrase, or Corolase. Different letters within the same raw material (roach or B. herring) indicate a statistically significant difference (*p* < 0.05).

Protein carbonyls and peroxide values in the studied protein isolates and hydrolysates are presented in Figure 2 and Figure 3. In general, isolates and hydrolysates from B. herring showed more signs of oxidation compared with roach, in terms of PV and protein carbonyls. B. herring, unlike roach, is a dark-muscled fish and hence contains more heme pigments, known to act as pro-oxidants in fish. The development of lipid oxidation has been observed even at a low lipid concentration of 0.1%, when pro-oxidants in fish blood were present [51]. Protein isolates produced by an alkaline pH shift from both raw materials showed high carbonyl values (14–33 nmol/mg protein), whereas protein hydrolysates showed moderate values (7.7–9.0 nmol/mg protein). Protein isolates produced using pH shift were subjected to a short heat treatment after precipitation (Supplementary Figure S1), which could explain the high carbonyl values. The heat treatment may have contributed to protein and lipid oxidation, indicated by the higher carbonyl values and PVs. In a study by Marmon and Undeland [52], protein carbonyls in herring protein separated by the alkaline pH-shift process were measured at a level of 2.5 nmol/mg protein and did not show a significant increase compared with the raw material. At the same time, however, lipid oxidation was observed to have significantly increased PV and TBARS. Nikoo et al. [22] reported the effect of different pretreatments on protein carbonyl values after 1, 2, or 3 h hydrolysis of rainbow trout by-product proteins using Alcalase. The lowest carbonyl value (<3 nmol/mg protein) was detected after 1 h of hydrolysis of the protein isolate without any pretreatments, but all values were below 4 nmol/mg protein, which is slightly less than observed in the present study.

In the case of both fishes, the highest PV was found in the lipids of the protein isolate produced using alkaline pH shift. Most previous studies have reported higher rates of oxidation for acidic pH shift compared with the alkaline one [18,53,54]. An acidic pH induces the unfolding of hemoglobin [55], making it more pro-oxidative, whereas as alkaline treatment may even have a protective effect compared with native hemoglobin [53]. However, a recent study [21] also reported significantly higher oxidation levels, indicated by higher malondialdehyde levels, for herring processed with alkaline compared with acidic pH shift, as was the case in the present study. The contradiction could be due to species-related differences in hemoglobin autoxidation. Due to the variations in the environmental conditions, such as water temperature, of different fishes, there are electrophoretically distinct forms of hemoglobin in fish that vary in their response to changes in pH [56]. The autoxidation of hemoglobin due to oxygen loss takes place in mildly acidic conditions (Root or Bohr effect), such as the precipitation pH 5.2 in the present study, which plays an important role in its pro-oxidativity [57]. In addition, in the pH-shift process the pH is adjusted in two sequential steps, and even if the precipitation pH is the same for both acidic and alkaline process, the previous solubilization pH is likely to alter the susceptibility of the system to lipid oxidation [58]. Nevertheless, findings from this and previous studies show that oxidation during the pH-shift process is a complex phenomenon and should be studied further. Increased lipid oxidation of alkaline protein isolates of roach and B. herring in the present study likely contributed to the lower levels of PUFAs observed.

Interestingly, the protein hydrolysates produced from roach using the three enzymes, Protamex, Neutrase, and Corolase, had similar PVs, but in the case of B. herring, treatment with Neutrase resulted in a higher PV in the hydrolysate. B. herring hydrolysate produced using Neutrase also showed a lower proportion of TAGs (Figure 1) and PUFAs (Table 3) compared with Protamex and Corolase, which was likely linked to oxidation. When Nile tilapia stored on ice for 0 or 18 days was hydrolyzed using Alcalase without antioxidants, the PV of the hydrolysate was 6-fold and 3-fold, respectively, higher compared with when hydrolysis was performed with the addition of antioxidants (Trolox and EDTA) [24]. When the muscle was stored on ice for 18 days prior to hydrolysis, the hydrolysate produced with antioxidants showed a similar PV to the hydrolysate produced from fresh muscle without antioxidants, highlighting the significance of both antioxidants and the use of fresh raw material for limiting lipid oxidation.

The volatiles of the raw materials and the protein isolates and hydrolysates were measured as another group of markers for oxidation. The most abundant volatiles found from roach raw material were propanal, 1-penten-3-ol, hexanal, heptanal, octanal, 3,5(*E*,*E*)-octadien-2-one and nonanal. All of these volatiles are formed from the oxidation of PUFAs. In the B. herring raw material, the most abundant volatiles were 2-methylbutanal, 3-methylbutanal, 1-penten-3-ol, 2,3-pentanedione, hexanal and heptanal. These Strecker aldehydes and volatile secondary lipid oxidation products were previously observed from fresh B. herring [37]. 

For the assessment of lipid oxidation in protein isolates and hydrolysates, seven volatile secondary lipid oxidation products were selected and semi-quantified as oxidation indicators. Propanal, 2-ethylfuran, 2-pentylfuran, 1-penten-3-ol, hexanal, 2(*E*)-hexenal, heptanal and 2,6(*E*,*E*)-nonadienal were chosen, since they were among the most abundant volatiles in the isolates and hydrolysates, and have been shown to be common in oxidized fish oil or other oils rich in n-3 or n-6 PUFAs [38,59]. Some of these volatiles have been previously proposed as oxidation indicators, e.g., Sajib and Undeland [60] recently suggested 2-pentylfuran as the best volatile marker for lipid oxidation in the production of silage from herring by-products, whereas 1-penten-3-ol and 2(*E*)-hexenal showed good potential to be used as indicator compounds for lipid oxidation in EPA- and DHA-rich products [38]. Most of the selected volatiles have also been associated with fishy and rancid odors [61].

**Figure 4:**
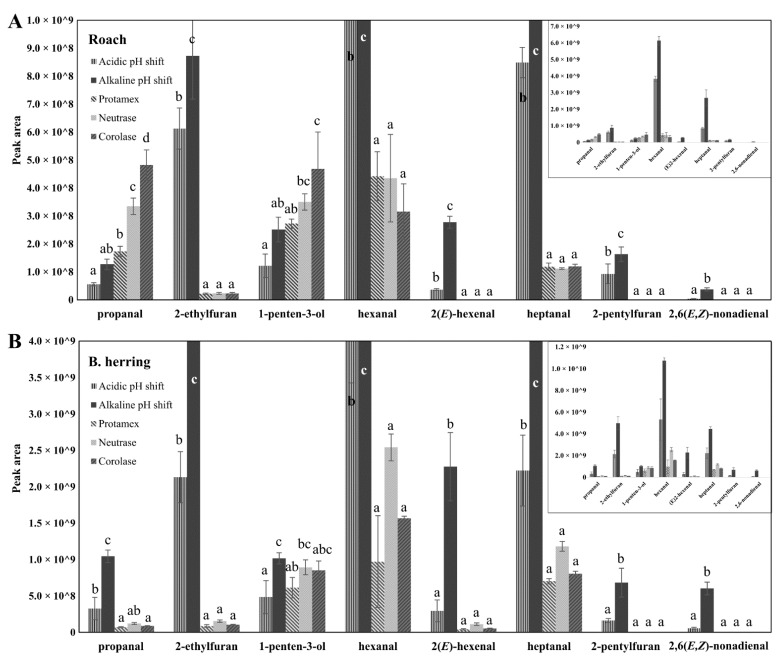
Peak areas of seven oxidation related volatiles, analyzed using headspace solid-phase microextraction-gas chromatography-mass spectrometry, in protein isolates from roach (**A**) and B. herring (**B**). Different letters indicate a statistically significant difference (*p* < 0.05) between samples.

The peak areas of the indicator volatiles showed a higher volatile formation in protein isolates and hydrolysates from B. herring compared with ones from roach (Figure 4). The higher content of DHA in isolates and hydrolysates from B. herring compared with the level in the isolates and hydrolysates from roach (Table 3) and an extra processing step (additional centrifugation; Supplementary Figure S1), as well as possible differences in levels of pro-oxidants, may have contributed to this difference in the development of oxidation status. The highest formation of lipid-oxidation-derived volatiles was seen in alkaline pH-shift-processed isolate, in the case of both fishes, followed by acid-treated isolate. Hexanal was the most abundant volatile followed by heptanal and 2-ethylfuran in the pH-shift isolates of both fishes. The ratio of 2-ethylfuran/hexanal was significantly higher in the pH-shift-processed isolates from B. herring compared with roach, which could be associated with the higher content of n-3 PUFAs in isolates from B. herring compared with the isolates from roach (Table 3), as 2-ethylfuran is produced from hydroperoxides of n-3 PUFAs through peroxyl radical cyclisation, while hexanal can be produced by scission of the lipid molecules on either side of the radical and is more associated with the lipid oxidation of n-6 PUFAs [59]. Phetsang et al. [54] reported the volatile composition of alkali- and acid-extracted protein isolates, surimi, and mince of farm-raised hybrid catfish (Clarias macrocephalus × Clarias gariepinus). Acid-extracted isolate showed the highest content of certain oxidation-related volatiles, such as octanal, nonanal and decanal, and also the highest TBARS values. In the present study, alkali-extracted protein isolate was more abundant in oxidation-related volatiles and was also the most oxidized based on PV and carbonyls.

2-Pentylfuran and 2,6(*E*,*E*)-nonadienal, and in the case of roach also 2(*E*)-hexenal, were only detected in the pH-shift-processed isolates (A and B) and not in the hydrolysates produced by Protamex, Neutrase and Corolase. This difference may be related to the higher lipid content (Table 1), the higher DHA content (Table 3) and the higher content of PLs (Figure 1) in the pH-shift-processed isolates compared with the enzymatically hydrolyzed samples.

In general, enzymatically produced hydrolysates showed lower volatile formation than the isolates produced by pH shift, indicating less lipid oxidation. In the case of roach, hexanal, 1-penten-3-ol and propanal, and in the case of B. herring, hexanal, 1-penten-3-ol, and heptanal were the most abundant volatiles in the hydrolysates. 1-Penten-3-ol was the only volatile, out of the selected volatile lipid oxidation indicators, that was equally or more abundant in the hydrolyzed samples than in the pH-shift samples in both fishes. 1-Penten-3-ol was one of the most abundant volatile compounds in all hydrolysates of Atlantic salmon by-products produced using varied process parameters, such as enzyme/substrate ratio and hydrolysis time [62]. In the roach hydrolysates, the peak area of propanal was also higher than in the acidic and alkaline isolates from roach. The intensities of volatile compounds derived from lipid oxidation were in line with PV and protein carbonyl measurements.

Based on PV and volatile data, protein isolates and hydrolysates produced from roach showed slightly less signs of lipid oxidation compared with the respective isolates and hydrolysates produced from B. herring (Table 1). The difference is most likely due to differences in lipid composition, an extra centrifugation step in the production of B. herring protein isolates and hydrolysates (Supplementary Figure S1), and possible differences in the levels of pro-oxidants, such as hemoglobin. In a recent study by Wu et al. [45], pH-shift-extracted protein isolates from herring and cod backbones were seen to be more susceptible to lipid oxidation than the product from salmon, and oxidation was found to correlate significantly with hemoglobin and iron contents. On the contrary, total content of PUFAs showed a low and non-significant correlation. In the present study, the proportion of PUFAs did not differ to a large extent between the isolates and hydrolysates of roach vs. B. herring (25.4–33.5 vs. 27.2–33.8%). B. herring raw material and protein isolates and hydrolysates extracted from it, however, contained 2–8 times as much PLs compared with roach raw material and protein isolates and hydrolysates. However, the most significant differences in the level of oxidation between isolates and hydrolysates was dependent on the fractionation method rather than the raw material. Overall, all protein isolates produced by pH shift were more oxidized than the enzymatically produced hydrolysates. The pH-shift-processed isolates from roach and B. herring were also reported to have a more intense rancid odor and flavor than enzymatically produced hydrolysates [29]. After the precipitation step, both alkali- and acid-solubilized proteins were subjected to a short heat treatment (Supplementary Figure S1), which may have accelerated the oxidation of lipids and proteins. In addition, the protein isolates (and hydrolysates) were finally freeze-dried, which as an additional processing step may further promote oxidation. In a previous study by Halldorsdottir et al. [63], freeze-drying increased the TBARS value of cod bone mince hydrolyzed with Protease P, but not when an antioxidative extract was present. Furthermore, since some fish-derived peptides have been found to possess antioxidative activities [5], it is possible that in the present study the formation of such peptides during enzymatic hydrolysis could have had a protective effect towards oxidation, and in part explain the difference in perceived oxidation compared with pH-shifted protein isolates.

Possible modifications to both enzymatic fractionation and pH-shift processing have been suggested to limit the oxidation during processing. Pre-treatments, such as washing and membrane separation prior to the hydrolysis of brownstripe red snapper, were efficient in lowering the myoglobin, PL, heme iron, and non-heme iron contents, and in reducing oxidation [11]. Abdollahi et al. [21] reported that lingonberry press cake, brown seaweed, and shrimp shells were efficient in reducing the lipid oxidation of pH-shift-processed protein isolates of herring and salmon, and our previous study showed that lingonberry and seabuckthorn press cake exhibited antioxidative effects in minced B. herring [37]. A few studies have also reported the production of fish protein isolate using pH shift and subsequent hydrolysis of the isolate to produce hydrolysate [11,22,26,28]. Prior protein extraction using the pH shift resulted in a decrease in PV and TBARS and increased liking scores for Nile tilapia and Indian mackerel hydrolysates when fortified in milk, when compared with hydrolysates produced directly from mince [26,28]. The results of the present study highlight the importance of controlling lipid and protein oxidation during the fractionation of roach and B. herring.

## 4. Conclusions

There were significant differences in the composition of protein isolates and hydrolysates of roach and B. herring, extracted using different processes. In the case of both raw materials, the protein isolates produced using pH shift showed higher essential to non-essential amino acid ratios compared with enzymatically processed hydrolysates. Both the fatty acid composition and the lipid classes varied among the protein isolates produced by different processes, which was likely associated with both the removal and oxidation of lipids during the processes. The acidic pH shift resulted in an increase in the proportion of phospholipids compared with the raw material and was accompanied by a high proportion of PUFAs, and especially DHA. Alkali-extracted B. herring protein isolate contained a significantly higher proportion of FFAs and less TAGs compared with the raw material, acidic isolate and hydrolysates, and was also the most oxidized as indicated by protein carbonyls, PV, and volatile oxidation products. Enzymatically extracted protein hydrolysates from both roach and B. herring produced using Protamex, Neutrase, or Corolase were relatively similar (within the same raw material) in terms of lipid composition and showed lower lipid and protein oxidation compared with the protein isolates obtained from pH-shift processes. In the case of B. herring, protein hydrolysate produced using Neutrase had a higher PV and showed higher levels of some oxidation-related volatile compounds compared with the hydrolysates produced with Protamex and Corolase. Contradictory to most previous studies, the alkali-processed protein isolates of both roach and B. herring were more oxidized compared with acid-treated isolates. The contradictory findings could be related to species-related differences in the pH-dependent pro-oxidativity of hemoglobin.

In terms of limiting oxidation, enzymatic hydrolysis was the more promising method for the valorization of proteins/peptides of both fish species, whereas extraction using pH shift was more favorable in terms of amino acid composition. However, the pH-shift process and enzymatic hydrolysis produce protein and peptide fractions with different molecular size and different functionalities, and since enzymatic fractionation produces hydrolysates, whereas pH shift does not hydrolyze the proteins to a large extent, the applications for protein isolates produced from these two processes are very different. Further research is needed to optimize the processing of B. herring and roach protein isolates and hydrolysates to limit the oxidation of lipids and proteins.

## Figures and Tables

**Table 1:** Protein and lipid contents/wet weight (as is) and dry weight (d.w.) in raw materials and processed protein isolates and hydrolysates from roach and B. herring, adapted from Nisov et al. [29].

Fish	Process	Protein (%, as is)	Protein, (%, d.w.)	Lipids (%, as is)	Lipids (%, d.w.)
Roach	Raw material	16.1 ± 0.1	64.9 ± 0.3	4.2 ± 0.0	17.0 ± 0.1
	Acidic pH shift	80.1 ± 1.0	83.0 ± 1.6	10.7 ± 0.5	11.1 ± 0.4
	Alkaline pH shift	70.8 ± 1.7	73.0 ± 2.0	18.8 ± 0.9	19.4 ± 0.9
	Protamex	80.4 ± 2.2	84.0 ± 1.5	5.5 ± 0.1	5.8 ± 0.1
	Neutrase	81.3 ± 1.6	85.1 ± 1.5	5.7 ± 0.2	6.0 ± 0.2
	Corolase	79.9 ± 1.6	83.6 ± 1.5	6.0 ± 0.1	6.3 ± 0.1
B. herring	Raw material	15.0 ± 0.0	63.1 ± 0.0	7.2 ± 0.1	29.9 ± 0.4
	Acidic pH shift	79.4 ± 0.7	80.4 ± 0.8	16.5 ± 1.0	16.7 ± 1.0
	Alkaline pH shift	76.0 ± 0.9	77.9 ± 0.7	16.4 ± 1.8	16.8 ± 1.8
	Protamex	87.2 ± 1.0	91.1 ± 1.2	3.1 ± 0.1	3.2 ± 0.1
	Neutrase	84.1 ± 1.3	87.9 ± 0.7	3.1 ± 0.2	3.3 ± 0.2
	Corolase	85.0 ± 1.4	88.7 ± 1.2	3.4 ± 0.2	3.5 ± 0.2

**Table 2:** Amino acids (mg/g protein) in protein isolates and hydrolysates from roach and B. herring, obtained using acidic or alkaline pH-shift process, or enzymatic fractionation using Protamex, Neutrase or Corolase. Different letters within the same row and raw material indicate a statistically significant difference (*p* < 0.05).

	Roach						B. Herring						
	Raw Material	Acidic pH Shift	Alkaline pH Shift	Protamex	Neutrase	Corolase	Raw Material	Acidic pH Shift	Alkaline pH Shift	Protamex	Neutrase	Corolase	FAO/WHO/UNU (2007)
Essential													
His	27.11 ± 2.72 ^b^	22.47 ± 2.77 ^a^	21.60 ± 2.78 ^a^	23.99 ± 1.93 ^ab^	24.26 ± 1.72 ^ab^	22.93 ± 2.15 ^a^	21.10 ± 0.42 ^c^	24.35 ± 1.33 ^d^	24.59 ± 0.76 ^d^	18.37 ± 0.65 ^a^	19.86 ± 0.48 ^bc^	19.38 ± 0.38 ^ab^	15
Ile	39.95 ± 2.47 ^b^	46.20 ± 0.71 ^c^	47.11 ± 1.58 ^c^	32.54 ± 0.62 ^a^	32.90 ± 1.02 ^a^	31.12 ± 0.74 ^a^	35.77 ± 0.81 ^b^	46.03 ± 1.74 ^c^	45.03 ± 0.77 ^c^	24.43 ± 0.63 ^a^	25.08 ± 0.32 ^a^	25.10 ± 0.26 ^a^	30
Leu	70.55 ± 3.48 ^b^	79.43 ± 0.74 ^c^	80.91 ± 2.53 ^c^	60.67 ± 1.00 ^a^	61.00 ± 1.43 ^a^	59.86 ± 1.42 ^a^	63.53 ± 1.11 ^b^	81.66 ± 2.97 ^c^	79.75 ± 2.15 ^c^	53.46 ± 0.82 ^a^	55.54 ± 0.49 ^a^	55.32 ± 0.66 ^a^	59
Lys	78.58 ± 14.00	85.99 ± 5.95	82.22 ± 12.93	77.61 ± 6.77	81.71 ± 5.54	73.94 ± 13.92	74.05 ± 4.93 ^ab^	79.92 ± 5.18 ^ab^	83.87 ± 3.86 ^b^	70.67 ± 7.81 ^a^	74.04 ± 5.71 ^ab^	74.68 ± 5.08 ^ab^	45
Met	25.76 ± 1.24 ^b^	28.28 ± 0.45 ^c^	29.01 ± 0.98 ^c^	21.66 ± 0.43 ^a^	21.93 ± 0.48 ^a^	21.25 ± 0.50 ^a^	24.62 ± 0.51 ^b^	29.94 ± 1.01 ^c^	30.01 ± 0.86 ^c^	20.71 ± 0.41 ^a^	21.02 ± 0.11 ^a^	21.31 ± 0.20 ^a^	22 *
Phe	36.63 ± 2.95 ^b^	40.51 ± 0.61 ^c^	41.81 ± 1.78 ^c^	29.62 ± 0.79 ^a^	29.84 ± 1.12 ^a^	29.19 ± 1.06 ^a^	32.73 ± 0.91 ^b^	37.99 ± 1.56 ^c^	38.27 ± 0.76 ^c^	22.91 ± 0.59 ^a^	23.14 ± 0.50 ^a^	23.75 ± 0.38 ^a^	38 **
Thr	38.14 ± 1.80 ^b^	41.28 ± 0.44 ^c^	42.36 ± 1.47 ^c^	33.93 ± 0.81 ^a^	34.01 ± 0.90 ^a^	33.71 ± 0.97 ^a^	36.24 ± 0.64 ^b^	42.14 ± 1.59 ^c^	42.20 ± 1.06 ^c^	31.25 ± 0.70 ^a^	31.91 ± 0.68 ^a^	31.83 ± 0.46 ^a^	23
Trp	10.03 ± 0.24 ^b^	11.71 ± 0.33 ^c^	13.51 ± 0.35 ^d^	7.20 ± 0.14 ^a^	7.42 ± 0.27 ^a^	6.73 ± 0.29 ^a^	9.04 ± 0.34 ^b^	11.84 ± 0.16 ^c^	12.30 ± 0.33 ^c^	6.06 ± 0.42 ^a^	5.96 ± 0.27 ^a^	6.35 ± 0.02 ^a^	6
Val	39.49 ± 1.88 ^b^	43.64 ± 0.34 ^c^	43.74 ± 1.54 ^c^	32.07 ± 0.78 ^a^	32.73 ± 0.67 ^a^	31.78 ± 0.76 ^a^	39.39 ± 0.72 ^b^	45.46 ± 1.77 ^c^	46.25 ± 1.22 ^c^	30.05 ± 0.70 ^a^	30.66 ± 0.41 ^a^	30.72 ± 0.51 ^a^	39
**Non-essential**													
Ala	48.70 ± 1.91 ^a^	50.12 ± 1.00 ^ab^	48.77 ± 1.71 ^a^	50.03 ± 1.38 ^ab^	49.32 ± 1.44 ^ab^	51.18 ± 1.40 ^b^	47.59 ± 0.43 ^a^	54.22 ± 2.11 ^c^	53.90 ± 1.51 ^c^	47.45 ± 0.72 ^a^	50.03 ± 0.72 ^b^	49.00 ± 0.81 ^ab^	
Arg	55.06 ± 2.82 ^a^	57.69 ± 0.73 ^b^	55.02 ± 2.38 ^a^	54.00 ± 1.41 ^a^	53.86 ± 0.96 ^a^	55.03 ± 1.18 ^a^	59.32 ± 0.33 ^b^	59.74 ± 2.51 ^b^	63.79 ± 1.96 ^c^	49.28 ± 1.24 ^a^	50.55 ± 1.03 ^a^	51.32 ± 1.07 ^a^	
Asn + Asp	85.34 ± 3.86 ^b^	91.71 ± 1.67 ^c^	92.25 ± 3.68 ^c^	79.01 ± 1.87 ^a^	79.29 ± 1.87 ^a^	78.87 ± 1.99 ^a^	72.24 ± 1.33 ^b^	86.84 ± 2.78 ^c^	87.96 ± 3.09 ^c^	67.77 ± 1.35 ^a^	69.40 ± 0.70 ^ab^	69.37 ± 1.17 ^ab^	
Cys ***	2.28 ± 1.10 ^c^	3.25 ± 0.46 ^d^	2.11 ± 0.43 ^bc^	1.25 ± 0.51 ^a^	1.44 ± 0.31 ^ab^	1.05 ± 0.15 ^a^	2.77 ± 0.19 ^c^	1.95 ± 0.82 ^b^	1.97 ± 0.39 ^b^	0.50 ± 0.20 ^a^	0.36 ± 0.07 ^a^	0.47 ± 0.21 ^a^	
Gln + Glu	124.56 ± 5.34 ^a^	134.62 ± 2.46 ^b^	133.14 ± 5.63 ^b^	123.09 ± 3.15 ^a^	122.62 ± 3.18 ^a^	125.50 ± 3.26 ^a^	104.98 ± 1.56 ^a^	131.52 ± 4.16 ^d^	125.50 ± 3.78 ^c^	114.16 ± 1.63 ^b^	119.02 ± 1.07 ^b^	117.60 ± 1.80 ^b^	
Gly	38.03 ± 1.46 ^b^	30.95 ± 1.27 ^a^	30.31 ± 2.44 ^a^	49.72 ± 2.02 ^c^	50.14 ± 2.25 ^c^	51.89 ± 2.11 ^c^	42.43 ± 2.43 ^b^	28.52 ± 1.20 ^a^	30.97 ± 0.73 ^a^	44.78 ± 1.86 ^bc^	46.71 ± 1.54 ^c^	47.02 ± 2.89 ^c^	
Hyp	5.29 ± 0.41 ^b^	1.31 ± 0.02 ^a^	1.27 ± 0.04 ^a^	10.87 ± 0.93 ^c^	10.63 ± 0.58 ^c^	11.91 ± 0.68 ^c^	6.74 ± 0.85 ^b^	1.95 ± 0.04 ^a^	2.05 ± 0.01 ^a^	8.70 ± 0.50 ^c^	9.46 ± 0.54 ^c^	9.07 ± 0.31 ^c^	
Pro	35.33 ± 1.53 ^b^	31.77 ± 0.58 ^a^	32.23 ± 1.26 ^a^	38.44 ± 1.45 ^c^	39.08 ± 0.82 ^c^	40.20 ± 0.73 ^c^	37.54 ± 1.34 ^b^	33.61 ± 1.78 ^a^	34.78 ± 1.18 ^a^	33.66 ± 1.12 ^a^	33.19 ± 0.86 ^a^	35.20 ± 1.15 ^ab^	
Ser	36.88 ± 1.74 ^bc^	37.35 ± 0.49 ^c^	37.23 ± 1.47 ^bc^	34.43 ± 0.89 ^a^	35.16 ± 0.82 ^a^	35.69 ± 0.98 ^ab^	33.74 ± 0.30 ^b^	37.04 ± 1.54 ^c^	37.80 ± 1.02 ^c^	31.61 ± 0.61 ^a^	32.46 ± 0.38 ^ab^	32.42 ± 0.57 ^ab^	
Tyr	32.55 ± 1.96 ^c^	37.14 ± 1.14 ^d^	38.40 ± 1.90 ^d^	24.40 ± 0.52 ^ab^	25.26 ± 0.78 ^b^	23.26 ± 0.64 ^a^	29.59 ± 0.49 ^b^	36.41 ± 1.41 ^c^	35.10 ± 1.38 ^c^	18.41 ± 0.43 ^a^	17.99 ± 0.41 ^a^	18.54 ± 0.29 ^a^	
**essential: non-essential**	0.79 ± 0.03 ^c^	0.84 ± 0.02 ^d^	0.85 ± 0.03 ^d^	0.69 ± 0.02 ^ab^	0.70 ± 0.01 ^b^	0.65 ± 0.03 ^a^	0.77 ± 0.02 ^b^	0.85 ± 0.01 ^c^	0.85 ± 0.01 ^c^	0.67 ± 0.01 ^a^	0.67 ± 0.01 ^a^	0.67 ± 0.01 ^a^	

* methionine + cysteine (16 + 6); ** phenylalanine + tyrosine; *** cysteine was only partially recovered with the method used.

**Table 3:** Fatty acids (mg/100 mg of total fatty acids) in roach, B. herring, and protein isolates and hydrolysates, obtained using acidic or alkaline pH shift process or enzymatic fractionation using Protamex, Neutrase, or Corolase. Different letters on the same row and within the same raw material indicate a statistically significant difference between samples (*p* < 0.05).

	Roach						B. Herring					
	Raw Material	Acidic pH Shift	Alkaline pH Shift	Protamex	Neutrase	Corolase	Raw Material	Acidic pH Shift	Alkaline pH Shift	Protamex	Neutrase	Corolase
Saturated												
14:0	2.26 ± 0.02 ^b^	1.94 ± 0.03 ^a^	2.32 ± 0.13 ^bc^	2.69 ± 0.01 ^e^	2.39 ± 0.03 ^cd^	2.44 ± 0.03 ^d^	5.37 ± 0.02 ^a^	5.51 ± 0.18 ^ab^	6.01 ± 0.21 ^d^	5.70 ± 0.03 ^bc^	5.89 ± 0.04 ^cd^	5.71 ± 0.05 ^c^
16:0	16.54 ± 0.11 ^a^	18.28 ± 0.02 ^b^	17.99 ± 0.24 ^b^	17.84 ± 0.16 ^b^	19.59 ± 0.49 ^c^	20.02 ± 0.46 ^c^	17.39 ± 0.06 ^a^	24.80 ± 0.13 ^e^	25.43 ± 0.27 ^f^	18.02 ± 0.26 ^b^	20.36 ± 0.11 ^d^	18.87 ± 0.21 ^c^
18:0	3.23 ± 0.06 ^a^	4.00 ± 0.04 ^c^	3.76 ± 0.14 ^b^	3.23 ± 0.04 ^a^	3.86 ± 0.17 ^bc^	3.99 ± 0.11 ^c^	1.65 ± 0.01 ^a^	2.69 ± 0.03 ^e^	2.72 ± 0.01 ^e^	1.78 ± 0.07 ^b^	2.14 ± 0.04 ^d^	1.93 ± 0.04 ^c^
others	1.13 ± 0.02 ^bc^	1.08 ± 0.02 ^ab^	1.06 ± 0.04 ^a^	1.12 ± 0.01 ^b^	1.17 ± 0.05 ^c^	1.17 ± 0.03 ^c^	1.13 ± 0.01 ^ab^	1.15 ± 0.02 ^bc^	1.19 ± 0.05 ^c^	1.10 ± 0.02 ^a^	1.16 ± 0.03 ^bc^	1.13 ± 0.01 ^ab^
Σ saturated	23.16 ± 0.15 ^a^	25.30 ± 0.05 ^b^	25.14 ± 0.27 ^b^	24.88 ± 0.21 ^b^	27.02 ± 0.73 ^c^	27.62 ± 0.63 ^c^	25.54 ± 0.07 ^a^	34.15 ± 0.28 ^e^	35.34 ± 0.52 ^f^	26.60 ± 0.35 ^b^	29.56 ± 0.12 ^d^	27.64 ± 0.24 ^c^
**Monounsaturated**												
16:1(n-7)	13.25 ± 0.10 ^a^	12.30 ± 0.06 ^a^	17.96 ± 1.59 ^b^	17.85 ± 0.12 ^b^	17.14 ± 0.59 ^b^	17.88 ± 0.52 ^b^	9.45 ± 0.11 ^e^	6.16 ± 0.27 ^a^	7.50 ± 0.21 ^b^	8.86 ± 0.10 ^d^	8.54 ± 0.04 ^c^	8.33 ± 0.06 ^c^
18:1(n-7)	5.22 ± 0.07 ^a^	5.49 ± 0.04 ^b^	5.53 ± 0.10 ^b^	5.39 ± 0.02 ^ab^	5.19 ± 0.16 ^a^	5.21 ± 0.27 ^a^	2.85 ± 0.04 ^a^	3.23 ± 0.04 ^c^	3.69 ± 0.04 ^d^	2.80 ± 0.05 ^a^	3.06 ± 0.06 ^b^	2.79 ± 0.07 ^a^
18:1(n-9)	22.88 ± 0.22 ^d^	21.72 ± 0.20 ^b^	20.67 ± 0.38 ^a^	21.75 ± 0.18 ^b^	22.42 ± 0.14 ^c^	22.38 ± 0.23 ^c^	25.62 ± 0.14 ^c^	19.73 ± 0.79 ^a^	22.84 ± 0.75 ^b^	26.34 ± 0.22 ^c^	26.17 ± 0.23 ^c^	25.98 ± 0.18 ^c^
20:1(n-9)	0.87 ± 0.02 ^ab^	0.97 ± 0.01 ^d^	0.87 ± 0.05 ^a^	0.91 ± 0.01 ^bc^	0.95 ± 0.02 ^cd^	0.93 ± 0.01 ^cd^	1.92 ± 0.02 ^c^	1.36 ± 0.07 ^a^	1.60 ± 0.08 ^b^	2.02 ± 0.01 ^d^	1.93 ± 0.02 ^c^	1.93 ± 0.03 ^c^
24:1(n-9)	0.17 ± 0.01 ^c^	0.23 ± 0.00 ^d^	0.22 ± 0.01 ^d^	0.11 ± 0.01 ^b^	0.09 ± 0.01 ^ab^	0.08 ± 0.02 ^a^	0.96 ± 0.01 ^a^	1.12 ± 0.01 ^d^	1.26 ± 0.02 ^e^	1.00 ± 0.02 ^b^	1.08 ± 0.00 ^c^	0.99 ± 0.02 ^b^
others	0.43 ± 0.02 ^a^	0.48 ± 0.02 ^ab^	0.56 ± 0.03 ^c^	0.45 ± 0.01 ^ab^	0.51 ± 0.07 ^bc^	0.46 ± 0.01 ^ab^	0.54 ± 0.01 ^ab^	0.50 ± 0.02 ^a^	0.56 ± 0.03 ^abc^	0.59 ± 0.01 ^bc^	0.62 ± 0.04 ^c^	0.61 ± 0.07 ^bc^
Σ monounsaturated	42.83 ± 0.17 ^b^	41.20 ± 0.25 ^a^	45.81 ± 1.10 ^c^	46.47 ± 0.11 ^cd^	46.29 ± 0.39 ^cd^	46.94 ± 0.42 ^d^	41.35 ± 0.25 ^c^	32.10 ± 1.15 ^a^	37.45 ± 1.07 ^b^	41.62 ± 0.27 ^c^	41.39 ± 0.24 ^c^	40.63 ± 0.28 ^c^
**Polyunsaturated**												
**n-3**												
18:3(n-3)	1.87 ± 0.05 ^d^	1.37 ± 0.03 ^c^	1.03 ± 0.11 ^ab^	1.15 ± 0.03 ^ab^	1.18 ± 0.14 ^b^	1.01 ± 0.11 ^a^	1.47 ± 0.02 ^c^	1.26 ± 0.02 ^b^	1.02 ± 0.03 ^a^	1.54 ± 0.01 ^d^	1.47 ± 0.03 ^c^	1.66 ± 0.03 ^e^
18:4(n-3)	0.61 ± 0.02 ^b^	0.48 ± 0.01 ^a^	0.60 ± 0.04 ^b^	0.68 ± 0.02 ^c^	0.63 ± 0.02 ^b^	0.62 ± 0.02 ^b^	1.63 ± 0.00 ^d^	0.96 ± 0.01 ^b^	0.74 ± 0.03 ^a^	1.66 ± 0.02 ^de^	1.40 ± 0.04 ^c^	1.69 ± 0.03 ^e^
20:3(n-3)	0.45 ± 0.01 ^c^	0.43 ± 0.01 ^c^	0.30 ± 0.04 ^ab^	0.34 ± 0.00 ^b^	0.31 ± 0.03 ^ab^	0.28 ± 0.03 ^a^	0.79 ± 0.01 ^c^	0.73 ± 0.01 ^b^	0.60 ± 0.01 ^a^	0.89 ± 0.01 ^e^	0.84 ± 0.01 ^d^	0.88 ± 0.01 ^e^
20:4(n-3)	0.72 ± 0.02 ^b^	0.66 ± 0.01 ^a^	0.73 ± 0.01 ^b^	0.83 ± 0.01 ^c^	0.75 ± 0.04 ^b^	0.73 ± 0.03 ^b^	1.16 ± 0.01 ^d^	0.93 ± 0.02 ^b^	0.70 ± 0.04 ^a^	1.21 ± 0.01 ^e^	1.03 ± 0.02 ^c^	1.15 ± 0.01 ^d^
20:5(n-3)	8.30 ± 0.12 ^c^	7.40 ± 0.04 ^b^	6.46 ± 0.16 ^a^	7.07 ± 0.06 ^b^	6.67 ± 0.31 ^a^	6.41 ± 0.27 ^a^	7.22 ± 0.04 ^c^	7.03 ± 0.31 ^c^	5.50 ± 0.46 ^a^	6.46 ± 0.04 ^b^	5.74 ± 0.05 ^a^	6.56 ± 0.02 ^b^
22:4(n-3)	0.03 ± 0.00 ^c^	0.03 ± 0.00 ^bc^	0.03 ± 0.00 ^bc^	0.04 ± 0.00 ^d^	0.03 ± 0.00 ^ab^	0.02 ± 0.00 ^a^	1.14 ± 0.02 ^e^	0.73 ± 0.02 ^b^	0.52 ± 0.02 ^a^	1.22 ± 0.01 ^f^	0.99 ± 0.02 ^c^	1.10 ± 0.02 ^d^
22:5(n-3)	2.31 ± 0.04 ^e^	1.98 ± 0.03 ^c^	1.95 ± 0.01 ^bc^	2.17 ± 0.03 ^d^	1.91 ± 0.01 ^ab^	1,88 ± 0,02	0.86 ± 0.03 ^d^	0.73 ± 0.03 ^b^	0.51 ± 0.04 ^a^	0.83 ± 0.01 ^cd^	0.70 ± 0.02 ^b^	0.81 ± 0.01 ^c^
22:6(n-3)	8.63 ± 0.17 ^c^	10.89 ± 0.23 ^d^	8.92 ± 0.14 ^c^	7.47 ± 0.07 ^b^	6.62 ± 0.26 ^a^	6.38 ± 0,31 ^a^	10.45 ± 0.05 ^b^	14.39 ± 1.08 ^c^	11.03 ± 1.02 ^b^	9.13 ± 0.14 ^a^	8.63 ± 0.25 ^a^	9.19 ± 0.21 ^a^
24:4(n-3)	0.02 ± 0.01 ^abc^	0.02 ± 0.01 ^c^	0.02 ± 0.00 ^bc^	0.01 ± 0.00 ^ab^	0.01 ± 0.00 ^ab^	0.01 ± 0.00 ^a^	1.10 ± 0.01 ^d^	0.72 ± 0.02 ^b^	0.52 ± 0.01 ^a^	1.17 ± 0.01 ^e^	0.95 ± 0.03 ^c^	1.07 ± 0.02 ^d^
24:5(n-3)	0.23 ± 0.00 ^c^	0.22 ± 0.00 ^b^	0.20 ± 0.01 ^a^	0.26 ± 0.00 ^d^	0.21 ± 0.01 ^a^	0.20 ± 0.00 ^a^	0.61 ± 0.06 ^b^	0.37 ± 0.09 ^a^	0.28 ± 0.12 ^a^	0.71 ± 0.11 ^b^	0.58 ± 0.12 ^b^	0.73 ± 0.07 ^b^
Σ n-3	23.15 ± 0.07 ^c^	23.50 ± 0.26 ^c^	20.25 ± 0.38 ^b^	20.01 ± 0.03 ^b^	18.31 ± 0.80 ^a^	17.55 ± 0.76 ^a^	26.44 ± 0.17 ^c^	27.87 ± 1.45 ^c^	21.44 ± 1.52 ^a^	24.82 ± 0.19 ^b^	22.34 ± 0.17 ^a^	24.83 ± 0.07 ^b^
**n-6**												
18:2(n-6)	4.85 ± 0.10 ^d^	3.70 ± 0.04 ^c^	3.32 ± 0.12 ^b^	3.72 ± 0.05 ^c^	3.24 ± 0.28 ^b^	2.93 ± 0.26 ^a^	3.80 ± 0.02 ^c^	3.26 ± 0.05 ^a^	3.39 ± 0.03 ^b^	4.01 ± 0.03 ^e^	3.92 ± 0.03 ^d^	4.05 ± 0.02 ^e^
20:2(n-6)	1.12 ± 0.02 ^cd^	1.17 ± 0.00 ^d^	0.95 ± 0.10 ^a^	1.02 ± 0.02 ^ab^	1.07 ± 0.02 ^bc^	1.03 ± 0.01 ^ab^	1.29 ± 0.01 ^c^	1.06 ± 0.03 ^b^	1.02 ± 0.01 ^a^	1.39 ± 0.01 ^e^	1.32 ± 0.01 ^d^	1.33 ± 0.01 ^d^
20:4(n-6)	4.11 ± 0.07 ^c^	4.37 ± 0.07 ^d^	3.77 ± 0.23 ^b^	3.09 ± 0.02 ^a^	3.21 ± 0.05 ^a^	3.10 ± 0.05 ^a^	0.74 ± 0.00 ^b^	0.89 ± 0.05 ^c^	0.76 ± 0.07 ^b^	0.66 ± 0.01 ^a^	0.67 ± 0.01 ^a^	0.68 ± 0.01 ^a^
22:2(n-6)	0.04 ± 0.00 ^b^	0.04 ± 0.00 ^b^	0.03 ± 0.00 ^a^	0.04 ± 0.00 ^b^	0.03 ± 0.00 ^ab^	0.03 ± 0.00 ^a^	0.71 ± 0.01 ^c^	0.56 ± 0.04 ^b^	0.52 ± 0.02 ^a^	0.76 ± 0.01 ^d^	0.69 ± 0.02 ^c^	0.71 ± 0.01 ^c^
others	0.74 ± 0.01 ^a^	0.73 ± 0.00 ^a^	0.73 ± 0.05 ^a^	0.77 ± 0.02 ^ab^	0.81 ± 0.03 ^b^	0.81 ± 0.04 ^b^	0.13 ± 0.00 ^d^	0.11 ± 0.00 ^b^	0.08 ± 0.00 ^a^	0.13 ± 0.01 ^d^	0.12 ± 0.00 ^c^	0.13 ± 0.00 ^d^
Σ n-6	10.85 ± 0.14 ^d^	10.01 ± 0.04 ^c^	8.80 ± 0.48 ^b^	8.64 ± 0.11 ^b^	8.37 ± 0.32 ^ab^	7.90 ± 0.29 ^a^	6.67 ± 0.04 ^c^	5.89 ± 0.06 ^b^	5.77 ± 0.08 ^a^	6.95 ± 0.04 ^d^	6.71 ± 0.03 ^c^	6.90 ± 0.03 ^d^
Σ polyunsaturated	34.00 ± 0.09 ^c^	33.50 ± 0.29 ^c^	29.05 ± 0.86 ^b^	28.65 ± 0.11 ^b^	26.69 ± 1.11 ^a^	25.44 ± 1.05 ^a^	33.10 ± 0.20 ^cd^	33.75 ± 1.39 ^d^	27.20 ± 1.59 ^a^	31.78 ± 0.19 ^c^	29.05 ± 0.14 ^b^	31.73 ± 0.05 ^c^
**Ratios**												
**unsaturated:saturated**	3.32 ± 0.03 ^c^	2.95 ± 0.01 ^b^	2.98 ± 0.04 ^b^	3.02 ± 0.03 ^b^	2.70 ± 0.10 ^a^	2.62 ± 0.08 ^a^	2.91 ± 0.01 ^f^	1.93 ± 0.02 ^b^	1.83 ± 0.04 ^a^	2.76 ± 0.05 ^e^	2.38 ± 0.01 ^c^	2.62 ± 0.03 ^d^
**n-3:n-6**	2.13 ± 0.03 ^a^	2.35 ± 0.02 ^c^	2.30 ± 0.08 ^c^	2.32 ± 0.03 ^c^	2.19 ± 0.03 ^ab^	2.22 ± 0.03 ^b^	3.97 ± 0.02 ^c^	4.74 ± 0.29 ^d^	3.71 ± 0.21 ^bc^	3.57 ± 0.04 ^ab^	3.33 ± 0.04 ^a^	3.60 ± 0.03 ^b^

## Data Availability

Not applicable.

## Supplementary Materials

The following are available online at https://www.mdpi.com/article/10.3390/foods11020230/s1, Figure S1: Schematic representation of the production of protein isolates and hydrolysates.
