# Supplementary material for: Quality of Protein Isolates and Hydrolysates from Baltic Herring (Clupea harengus membras) and Roach (Rutilus rutilus) Produced by pH-Shift Processes and Enzymatic Hydrolysis
Source: Foods. 2022 Jan 15;11(2):230. doi: 10.3390/foods11020230 (PMC8775156; doi:10.3390/foods11020230)
Supplement: Supplementary file 1 [file foods-11-00230-s001.zip › foods-1521947-supplementary.pdf]

Supplementary Material

The supporting information is provided: (1) Schematic representation of the production of protein isolates and hydrolysates (**Figure S1**).

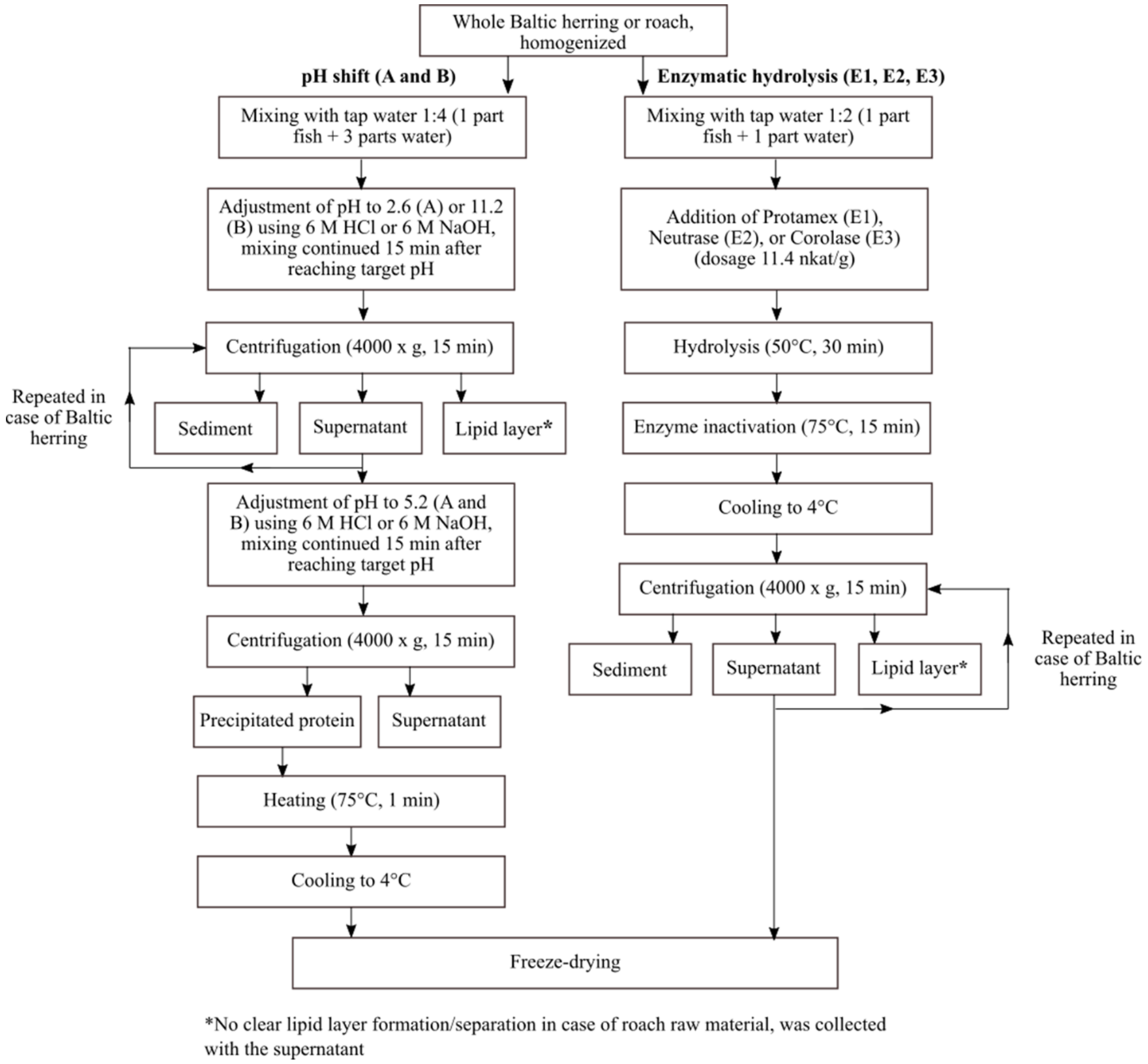

Figure S1. Schematic representation of acidic (A) and alkaline (B) pH shift process and enzymatic hydrolysis using Protamex (E1), Neutrase (E2), or Corolase (E3), as previously reported by Nisov et al. [20]. Unless stated otherwise, processing was conducted at 4ºC and under continuous mixing (75 rpm).
